# Patterns of Service Use in Intensive Case Management: A Six Year Longitudinal Study

**DOI:** 10.1007/s10488-022-01198-y

**Published:** 2022-05-16

**Authors:** Philippe Golay, Charles Bonsack, Benedetta Silva, Guillaume Pauli, Eva de Boer, Stéphane Morandi

**Affiliations:** 1 Service of Community Psychiatry, Department of Psychiatry, Lausanne University Hospital and University of Lausanne, Consultations de Chauderon, Chauderon 18, 1003 Lausanne, Switzerland; 2 Service of General Psychiatry, Treatment and Early Intervention in Psychosis Program (TIPP-Lausanne), Lausanne University Hospital and University of Lausanne, Lausanne, Switzerland; 3 Institute of Psychology, Faculty of Social and Political Science, University of Lausanne, Lausanne, Switzerland; 4 Cantonal Medical Office, Department of Health and Social Action (DSAS), General Directorate for Health of Canton of Vaud, Avenue des Casernes 2, 1014 Lausanne, Switzerland

**Keywords:** Assertive community treatment, Intensive case-management, Psychiatric hospitalization, Patterns, Service use

## Abstract

An Intensive Case Management (ICM) intervention has been developed in Lausanne, Switzerland. It aims to promote access to care for people with severe mental disorders who have difficulties to engage with mental health services because of the severity of their disorders and/or their marginality. ICM embrace components of assertive community treatment and critical time intervention. It is time limited and focused on critical phases of recovery. The goal of this study was to examine the heterogeneity of service use patterns of people who required ICM interventions and identify differences in patterns of duration and timing of the intervention. Records of 471 patients from the Department of Psychiatry of Lausanne University Hospital for whom the ICM team intervention was requested were analysed over a 6 year period with discrete sequential-state analysis. Trajectories could be split between six meaningful clusters including service light use and critical time intervention (58.0%), transition to long-term regular ambulatory-care (11.3%), partial transition to ambulatory care (14.4%), alternative to hospitalization (10.4%), continued ICM (4.9%) and long hospital stays (1.1%). Diagnoses of substance abuse were overrepresented among heavy users and diagnoses of schizophrenia were the most frequent diagnostic overall. Profiles of service use for ICM patients were very diverse. Long term interventions were frequently not necessary. A time-limited intervention was likely sufficient to stabilize the situation and/or engage the patient in care. A small number of situations required a sustained and long-term investment and did not always allowed for a reduction in the need for hospitalization. A general reflection on alternatives to hospitalization must be pursued, in particular for these patients.

## Introduction

Intensive Cases Management (ICM) ameliorates many outcomes relevant to people with severe mental illness (Dieterich et al., 2017). Compared to standard care, ICM may reduce hospitalization and increase retention in care. It also globally improves social functioning. ICM is particularly interesting for the subgroup of people with a high level of hospitalisation. This study was interested in the service sequences of ICM and the service patterns among different clinical characteristics of service users.

Assertive Community Treatment (ACT) is one of the first ICM models that was developed in the 1970s, at the height of the deinstitutionalization movement in psychiatry (Burns & Firn, 2002). This approach aims to offer community care to people suffering from severe mental disorders on a time-unlimited basis. ACT is provided by multidisciplinary teams with a low patient-to-staff ratio in order to offer intensive medical as well as social support to participants. ACT emphasis on home visit and other community interventions. Subsequently, the original ACT intervention has sometimes been adapted to suit local contexts. In Holland, the flexible ACT (FACT) model recommends to adjust the intensity of the intervention and the involvement of the team members according to patients’ needs (Nugter et al., 2016; Veldhuizen, 2007). Some models of ICM, such as the Critical Time Intervention (CTI), offer rather short-term interventions, focusing on critical periods, as for instance moving into housing after a period of homelessness or returning to the community after imprisonment (Herman & Mandiberg, 2010). More generally, the challenges related to the ICM are to be able to adapt care to meet the patients’ needs and to enable them to lead a satisfactory life in the community, despite the symptoms of the illness, according to the concepts of recovery. In addition, it is a question of offering an efficient intervention, based on scientific evidence, which complements the services provided by other mental health professionals.

In 2001, an ICM programme (in French Suivi Intensif dans le milieu-SIM), was developed in Lausanne, Switzerland, an urban area of 265,000 inhabitants (Conus et al., 2001; Morandi et al., 2017). The ICM was targeted at difficult-to-engage patients with severe and persistent mental disorders, high psychiatric care utilization, repeated hospitalizations or lack of connection to outpatient psychiatric care (Bonsack et al., 2005). Patients are referred to the programme by third parties: hospital teams, treating physicians, other outpatient teams, especially early intervention teams (Alameda et al., 2016), but also by family members, social services, guardians, the police or judges. The intervention combined the Assertive Community Treatment (ACT) (Burns et al., 2006) and the Critical Time Intervention (CTI) (Herman, 2014) methodologies.

The defining characteristics of ACT is that case managers and psychiatrists are able to provide home visits when needed. The caseload is limited to 20 patients per full-time professional to allow the case managers to spend more time with each person and to intensify the follow-up during crisis periods. ACT is multidisciplinary and not exclusively focused on the illness. Each professional can discuss and provide specific help on a wider range of issues, such as housing or income (Alameda et al., 2016; Morandi et al., 2017). These characteristics of ACT are key elements that contribute to clients’ satisfaction and promote their engagement with care (Priebe et al., 2005). ACT is considered to be an effective alternative to hospitalisation for people who are difficult to engage in care (Dieterich et al., 2017). The benefit of ACT also appears to be less in Europe than in the US, probably due to a different organisation of the health system, which in Europe offers other outpatient alternatives to psychiatric care (Killaspy et al., 2006).

For these reasons, but also because of a lack of resources, the ICM programme in Lausanne differed from ACT in several aspects. The team was only available Monday to Friday between 8 am and 6 pm. That means that during nights and weekends, clients could be referred to the local psychiatric emergency department. The collaboration developed with the emergency services makes it possible to respond to eventual emergency situations. Also, even if situations were regularly discussed among team members, each client was followed by a specific case manager (and by the ICM psychiatrist when no other doctor was involved in the situation). Finally, the team members only delivered services that other professionals could not provide, such as intensive home visits or practical help for time consuming administrative procedures. Therefore, a collaboration with social and medical services capable of providing assistance to the patient is established from the beginning of the intervention and the transition to these services is made as soon as possible.

The ICM programme also borrowed elements from the CTI model (Herman, 2014): the intervention was time-limited and focused on critical or transitional periods; it aimed to engage clients with other services through a smooth process; during the programme, it offered a psychological as well as a practical help adapted to client’s needs; client’s resources and limitations were assessed in vivo and practical solutions proposed. CTI favours intensive case management interventions at critical moments such as discharge from psychiatric hospital or prison.

The ICM model developed in Lausanne is therefore a form of flexible intensive case management, which is personal recovery rather than chronicity oriented with no lifetime follow-up. The aim is to promote access to standard care as soon as possible but also promote the development of a fulfilling life and a positive sense of identity based on hope and self-determination (Huguelet, 2007).

The goal of this study was to examine the heterogeneity of service use patterns of people who required ICM interventions. The aim was to identify differences in patterns of duration and timing of the intervention, thus in order to determine different forms of intensive case management adapted to the specific needs of patients’ sub-populations. We hypothesized that there would be high heterogeneity in the use of the service and important differences with regards to long or short term follow-up by the ICM team. In the year preceding the intervention, the person may or may not be a high-user of acute psychiatric hospital care. Indeed, on the one hand, some people may be referred to ICM team because they avoid care or have no access to the health system. On the other hand, other people may be regularly admitted to hospital or to emergency services or they may have long-lasting hospital stays before the intervention of the ICM team. In the 5 years following the intervention, intensive follow-up may be either short or long-term. A proportion of patients, yet to be determined, may recover and return to standard care. Moreover, following ICM, the use of inpatient and emergency care may decreases for high-users of acute psychiatric care but it could also increase for non-users of psychiatric care due to an easier access to care for previously marginalised people. The proportions of these different patterns are currently unknown in our setting.

## Materials and Methods

### Procedure and Participants

The catchment area of the ICM team counted about 250,000 inhabitants, Lausanne town and its proximal region. The inclusion criteria was patients for whom the ICM team intervention was requested starting from February 2011 to November 2020. All patients for which service use data was available starting one year prior to the ICM team request and up to 5 years later were included in this study. There was no exclusion criteria.

The Department of Psychiatry of Lausanne University Hospital is the only provider of hospitalization and ICM care for the area. Coded IDs from patients were listed and service use data including public psychiatric outpatient care as well as possible deaths were extracted from institutional records. We counted all services performed in the presence or absence of the patient. This includes visits and telephone calls.

Outpatient follow-up by doctors and other self-employed carers was not included due to the lack of data available for these services. Limited clinical data such as presence of personality disorder, presence of substance use disorder, Health of the Nation Outcome Scale (HoNOS) scores (Pirkis et al., 2005; Wing et al., 1998) and main diagnostic were also available if an hospitalization occurred. Data of the hospitalization nearest to the introduction of ICM was selected considering this could be the most representative of the patients’ situation at the time of the ICM team request.

For each patient, service use data was aggregated into a string of 313 digits representing service use during 6 years (1 year before ICM and 5 years after) with a time resolution of one week. For each week, a different number was used to represent each state (no service provided, hospitalization, ICM ambulatory care, other ambulatory care or death). If several services took place during the same week, we prioritized hospitalization, then ICM ambulatory Care and then other ambulatory care so as not to increase the number of different states excessively. This prioritisation was chosen based on the intensity of care provided by these three services and the level of care needs of their corresponding patients. Hospitalisation was considered as the most intensive level of care, followed by ICM and both responding to high-needs patients. Other ambulatory care was considered has standard intensity care for standard-needs patients. Access to the existing routine institutional records data was granted by the Human Research Ethics Committee of the Canton of Vaud (protocol #2016-00768).

### Statistical Analysis

The outline of the analysis plan was threefold: (1) we analysed all parents’ service sequences and computed their similarity, (2) we used cluster analysis to identify service use patterns and (3) we compared the clinical characteristics among the clusters.

The service use sequences were analysed with the TraMineR package for R which allowed discrete sequential-state/event analysis (Gabadinho et al., 2011). Sequences were analysed as states and the dissimilarity of each pair of sequences was computed using the *Optimal Matching* algorithm which generates edit distances that are the minimal cost, in terms of insertions, deletions and substitutions, for transforming one sequence into another (Gabadinho et al., 2009a). A cluster analysis using Ward’s method was performed on this dissimilarity matrix in order to group similar individual trajectories. Clusters were interpreted using visualization and statistical tools within the TraMineR package (Gabadinho et al., 2009b). We extracted an increasing number of clusters and the final number of clusters was determined on the basis on interpretability (Golay et al., 2019a).

Given the relatively large number of cluster (6), we used a Bayesian model comparison approach to examine clinical data between clusters. This represents an elegant alternative to the classic problem of multiple comparisons and allows evaluating the support for the null hypothesis (Golay et al., 2019b, 2020; Noël, 2015). The first model was the homogeneous model (1, 2, 3, 4, 5, 6), stating that the six groups did not differ and were issued from the same distribution. It corresponds to the null hypothesis in the classical statistical testing framework. Another model was the heterogeneous model: (1), (2), (3), (4), (5), (6) (i.e., all the groups were different from each other and were issued from six different distributions). All other possible combinations, which adds up to 203—for instance (1, 2, 3), (4, 5, 6) or (1, 2, 3), (4, 5), (6)—were also estimated. For continuous variables, the best possible Gaussian model (μ, σ2) was determined by using the Bayesian information criterion (Schwarz, 1978). For nominal variables, the best multinomial model was determined using the exact likelihood with a uniform prior on all parameters (Noël, 2015). An equal prior probability of 1/203 was assumed for all models so that no model was favoured. The Bayes factor was also computed (Kass & Raftery, 1995) and provided a comparison between the best model and the homogenous model. A Bayes factor of 4 indicates that the best model was 4 times more likely to be true than the homogenous model. Values over 3 are generally considered sufficiently important to favour one model over another (Jeffreys, 1961; Wagenmakers et al., 2011). All statistical analyses were performed using IBM SPSS, version 27, the AtelieR package for R (Noël & AtelieR, 2013), the TraMineR package for R (Gabadinho et al., 2009b) and the Bayes R2STATS group models calculator (Noël, 2018).

## Results

The ICM team intervention was requested for 887 patients between February 2011 and November 2020. Service use data starting one year prior to the ICM team request was available for all of them. 416 patients were excluded because the ICM intervention was too recent to have a 5 year follow-up. The records of 471 patients were analysed. Mean age was 35.3 (SD = 12.86) years old and 56.9% were men. Main diagnostic for patients with hospitalization data were the following: Schizophrenia (45.8%), Depression (13.0%), Drug use (11.3%), Alcohol use (10.2%), Personality disorder (7.4%), Mania (6.0%), Anxiety and stress related disorder (3.2%), Dementia (1.8%) and Behavioural syndromes associated with physiological disturbances and physical factors (1.4%). 39.1% of patients suffered from a substance use disorder and 18.0% from a personality disorder.

**Fig. 1:**
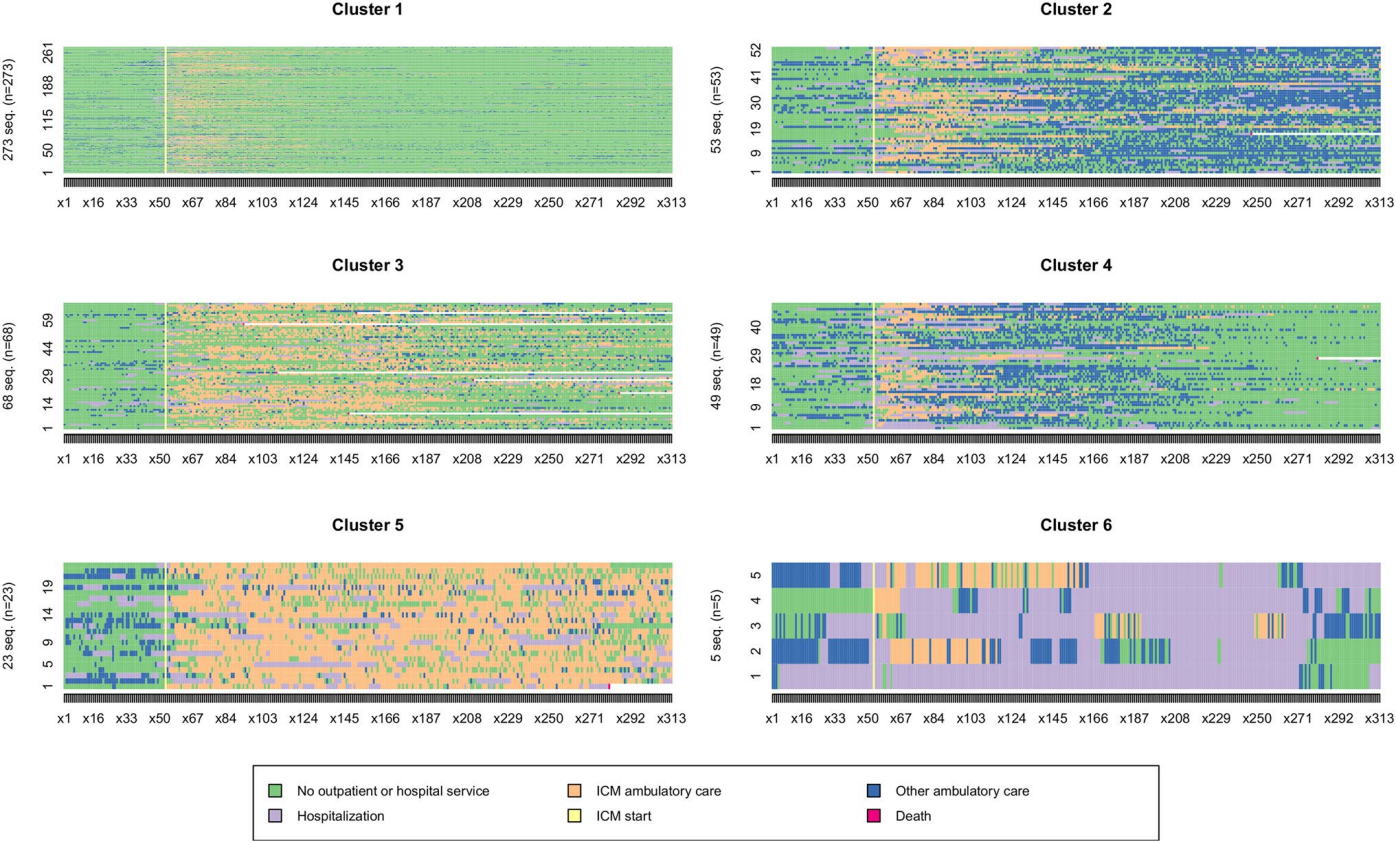
Clusters’ characteristics

Six meaningful clusters were identified: Cluster 1 “*Critical time intervention*” (N = 273; 58.0%) corresponds to patients with light use of services. In this cluster, the intervention pattern is light, transient and of short duration. It corresponds to interventions in critical periods for users who can then quickly return to standard outpatient care. Cluster 2 “*Transition to long-term ambulatory care*” (N = 53; 11.3%) likely corresponds to patients who transitioned from ICM to intense use of regular ambulatory care (Fig. 1).

**Fig. 2:**
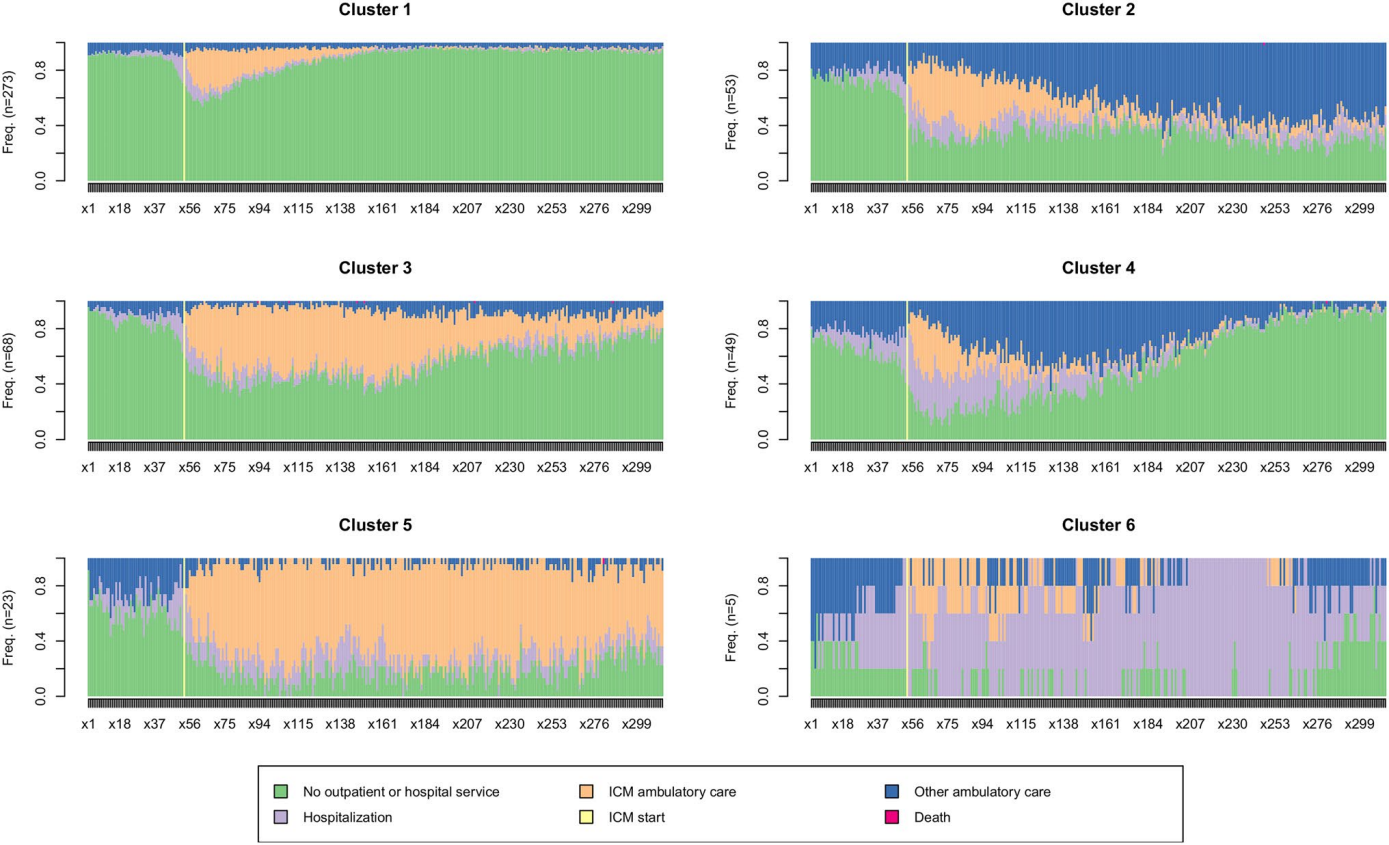
Clusters’ state distribution

The state distribution showing less frequent use of ICM over time in favour of other ambulatory care is presented on Fig. 2. Cluster 3 “*Partial transition to ambulatory care*” (N = 68; 14.4%) corresponds to patients for which ICM services were introduced in a context of frequent hospitalization which diminished over time after ICM services were introduced. No evident increase in regular ambulatory care is to be seen. Cluster 4 “*Alternative to hospitalization*” (N = 49; 10.4%) corresponds to patients for which ICM services were introduced in a context of intense hospitalizations and transitioned from intense use of both ICM and hospital to regular ambulatory care. Regular ambulatory care then evolved to very light use of services over time. Cluster 5 “*Continued ICM*” (N = 23; 4.9%) corresponds to patients with an extended and stable use of ICM services over time with few hospitalizations. Cluster 6 “*Long hospital stays*” (N = 5; 1.1%) corresponds to patients with very long hospital stays despite ICM intervention.

**Fig. 3:**
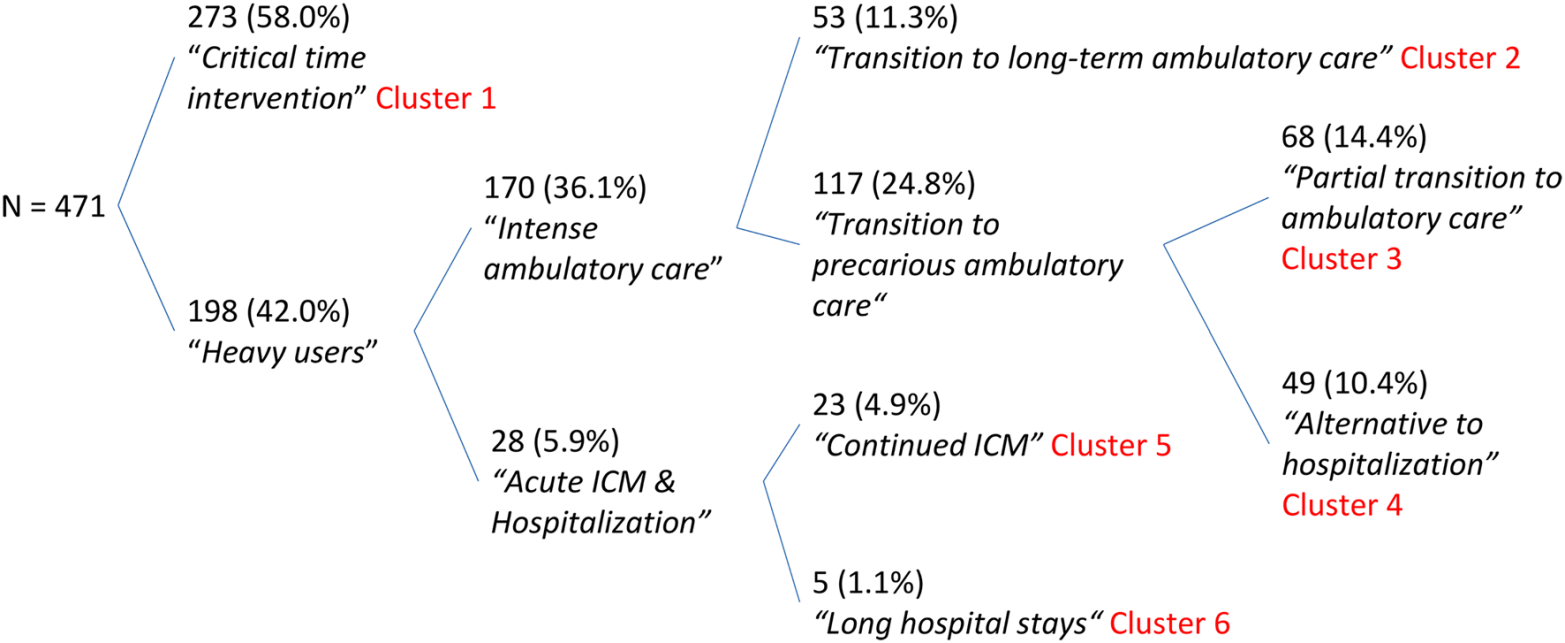
Clusters’ extraction sequence

The sequence of extraction gave us another insight on the characteristics of the groups (Fig. 3). Firstly, trajectories were split into heavy-users (198; 42.0%) and light users/Critical time intervention (273; 58%; Cluster 1). The heavy-users intervention pattern resembles that described for intensive case management in the broad sense (an intensive intervention in the living environment, of varying intensity and duration). It could be further split into patient with an intense use of other ambulatory care which resemble to an intensive case management pattern and patients with an intense use of ICM services and hospitalizations which is more prototypical of an ACT-type intervention. Patients with intense other ambulatory care could be classified into two distinct clusters whether ICM use consisted of a transitional period to other ambulatory care (Cluster 2) or ICM potentially allowed a transition to a more precarious ambulatory care. This group could be split in two groups defined by partial transition to ambulatory care (Cluster 3) or where ICM potentially consisted of an alternative to hospitalization (Cluster 4). Patients with intense ICM and Hospitalization use could be split into two groups mainly based on the intensity of hospital use: the first group (Cluster 5) had very intense ICM with relatively little hospitalizations and the second group corresponded to patients with very long hospital stays (Cluster 6) despite ICM intervention. The extraction of a seventh cluster divided cluster one into two similar groups, whose distinguishing feature was a slight difference in the overall intensity of service use, with no difference in temporal dynamics. For the sake of parsimony, no further clusters were extracted and the six clusters solution was retained.

A total of nine patients died during the follow-up, six of which appeared in cluster 3. Comparison of other patients characteristics are presented in Table 1. Clusters were homogeneous relative to age, gender, aggressive behaviour, physical illness or disability problems, hallucinations and delusions, mood, daily living and occupational activities and the amount of severe HoNOS problems.

**Table 1:** Patients’ characteristics in the different clusters

	C1—N = 273 (available hosp. data: N = 131)	C2—N = 53 (available hosp. data: N = 46)	C3—N = 68 (available hosp. data: N = 45)	C4—N = 49 (available hosp. data: N = 40)	C5—N = 23 (available hosp. data: N: = 17)	C6—N = 5 (available hosp. data: N: = 5)	Best model^a^	Bayes Factor against null hypothesis^b^	Probability of the model to be true^c^
Age, mean (SD)	36.3 (13.3)	33.1 (11.2)	34.0 (12.7)	33.6 (11.4)	34.3 (14.0)	39.0 (13.0)	(1, 2, 3, 4, 5, 6)	1.0000	.1488
Gender, male % (N)	54.9 (150)	58.5 (31)	66.2 (45)	61.2 (30)	43.5 (10)	40.0 (2)	(1, 2, 3, 4, 5, 6)	1.0000	.0389
*Hospitalization data*									
Presence of substance use disorder, % (N)	35.1 (46)	43.5 (20)	42.2 (19)	37.5 (15)	41.2 (7)	80.0 (4)	(1, 2, 3, 4, 5), (6)	2.4332	.0813
Presence of personality disorder, % (N)	16.0 (21)	15.2 (7)	15.6 (7)	30.0 (12)	17.6 (3)	20.0 (1)	(1, 2, 3, 5, 6), (4)	1.4230	.0664
*Main diagnostic, % (N)*									
Dementia	1.5 (2)	0.0 (0)	2.2 (1)	5.0 (2)	0.0 (0)	0.0 (0)	(1, 2, 3, 4,5), (6)	1.1882	.5326
Alcohol use	7.6 (10)	17.4 (8)	8.9 (4)	10.0 (4)	11.8 (2)	20.0 (1)			
Drug use	10.7 (14)	8.7 (4)	11.1 (5)	15.0 (6)	5.9 (1)	40.0 (2)			
Schizophrenia	45.8 (60)	47.8 (22)	48.9 (22)	42.5 (17)	52.9 (9)	0.0 (0)			
Mania	6.1 (8)	6.5 (3)	6.7 (3)	5.0 (2)	5.9 (1)	0.0 (0)			
Depression	16.8 (22)	6.5 (3)	8.9 (4)	12.5 (5)	11.8 (2)	20.0 (1)			
Anxiety and stress related disorder	3.8 (5)	2.2 (1)	6.7 (3)	0.0 (0)	0.0 (0)	0.0 (0)			
Behavioural syndromes associated with physiological disturbances and physical factors	0.8 (1)	4.3 (2)	2.2 (1)	0.0 (0)	0.0 (0)	0.0 (0)			
Personality disorder	6.9 (9)	6.5 (3)	4.4 (2)	10.0 (4)	11.8 (2)	20.0 (1)			
*Honos scores, % of severe problems (score 3 or 4) (N)*									
1 Overactive, aggressive, disruptive or agitated behaviour	27.3 (33)	37.8 (14)	37.5 (15)	24.2 (8)	35.7 (5)	40.0 (2)	(1, 2, 3, 4,5,6)	1.0000	.0415
2 Non-accidental self-injury	13.6 (16)	0.0 (0)	9.8 (4)	11.8 (4)	0.0 (0)	0.0 (0)	(1, 3, 4), (2, 5, 6)	13.2507	.1834
3 Problem drinking or drug-taking	37.0 (40)	33.3 (10)	41.5 (17)	34.4 (11)	57.1 (8)	100.0 (5)	(1, 2, 3, 4, 5), (6)	18.8065	.0962
4 Cognitive problems	8.7 (9)	18.8 (6)	7.9 (3)	10.3 (3)	21.4 (3)	25.0 (1)	(1, 3, 4), (2, 5, 6)	1.2423	.0577
5 Physical illness or disability problems	12.6 (14)	2.9 (1)	10.0 (4)	11.8 (4)	14.3 (2)	20.0 (1)	(1, 2, 3, 4, 5, 6)	1.0000	.0810
6 Problems associated with hallucinations and delusions	50.9 (58)	55.6 (20)	51.3 (20)	37.9 (11)	42.9 (6)	40.0 (2)	(1, 2, 3, 4, 5, 6)	1.0000	.0460
7 Problems with depressed mood	35.6 (42)	43.8 (14)	30.8 (12)	48.5 (16)	28.6 (4)	40.0 (2)	(1, 2, 3, 4, 5, 6)	1.0000	.0400
8 Other mental and behavioural problems	50.0 (54)	35.3 (12)	46.2 (18)	60.0 (18)	36.4 (4)	25.0 (1)	(1, 3, 4), (2, 5, 6)	1.4604	.0347
9 Problems with relationships	48.2 (53)	44.4 (16)	51.2 (21)	50.0 (16)	41.7 (5)	80.0 (4)	(1, 2, 3, 4, 5), (6)	1.1899	.0613
10 Problems with activities of daily living	33.6 (37)	40.0 (14)	29.7 (11)	35.5 (11)	27.3 (3)	60.0 (3)	(1, 2, 3, 4, 5, 6)	1.0000	.0528
11 Problems with living conditions	36.3 (41)	41.2 (14)	43.2 (16)	33.3 (10)	50.0 (6)	75.0 (3)	(1, 2, 3, 4, 5), (6)	1.4055	.0519
12 Problems with occupation and activities	48.1 (52)	54.5 (18)	57.9 (22)	51.6 (16)	50.0 (6)	50.0 (2)	(1, 2, 3, 4, 5, 6)	1.0000	.0544
13 Problems with psychiatric medication (French Honos extra item)	50.9 (55)	69.7 (23)	43.2 (16)	53.1 (17)	46.2 (6)	80.0 (4)	(1, 3, 4, 5), (2, 6)	4.2226	.0681
Total of severe Honos items, Mean (SD)	3.8 (2.4)	3.5 (2.8)	4.0 (2.6)	3.6 (2.8)	3.4 (2.9)	6.0 (3.0)	(1, 2, 3, 4, 5, 6)	1.0000	.2239

^a^On the basis of the BIC coefficient

^b^Bayes factor comparing the best model with the homogeneous model (1, 2, 3, 4, 5, 6)

^c^Among all 203 possible models

Several differences between clusters could be highlighted. Patients were more likely to have a substance use disorder, problems with relationships and with living conditions in cluster 6. The most frequent diagnostic across all clusters was schizophrenia (48.8%) but presence of personality disorder were more likely in cluster 4.

Non-accidental self-injury and other mental and behavioural problems were also more likely in clusters 1, 3, and 4 while cognitive problems were more likely in cluster 2, 5 and 6. Finally, problems with psychiatric medication were more likely in clusters 2 and 6.

A comparison of service use between clusters is provided in Table 2. Patients of cluster 6 were among the most heavy-users of hospitalization although given their small number it represented only 11.34% of the total service use. Patients from cluster 2 and 4 were among the most heavy-users of general ambulatory care. Finally, patients from cluster 5 were among the patients with the most heavy-use of ICM ambulatory care. Nevertheless, patients of cluster 3, because they were more numerous, requested more ICM overall.

**Table 2:** Service use: comparison between clusters

	n	% total	*Hospitalization weeks*				*General Ambulatory care weeks*				*ICM ambulatory care weeks*			
			*Sum*	*% total*	*Per patient*	*% total Per patient*	*Sum*	*% total*	*Per patient*	*% total per patient*	*Sum*	*% total*	*Per patient*	*% total per patient*
Cluster 1 “Critical time”	273	57.96	2098	25.37	7.68	2.45	3789	22.97	13.88	4.45	4224	25.37	15.47	4.41
Cluster 2 “Transition”	53	11.25	1423	17.21	26.85	8.54	6499	39.39	122.62	39.30	2034	12.22	38.38	10.95
Cluster 3 “Partial transition”	68	14.44	1188	14.37	17.47	5.56	1507	9.13	22.16	7.10	5450	32.73	80.15	22.86
Cluster 4 “Alternative”	49	10.40	1704	20.61	34.78	11.06	3894	23.60	79.47	25.47	961	5.77	19.61	5.59
Cluster 5 “Continued ICM”	23	4.88	918	11.10	39.91	12.70	563	3.41	24.48	7.85	3827	22.99	166.39	47.46
Cluster 6 “Long stays”	5	1.06	938	11.34	187.60	59.69	247	1.50	49.40	15.83	153	0.92	30.60	8.73
Total	471	100.00	8269	100.00	17.56	100.00	16,499	100.00	35.03	100.00	16,649	100.00	35.35	100.00

## Discussion

Profiles of service use for ICM patients were very diverse. Discrete sequential-state statistical analysis allowed the identification of six clinically meaningful service use patterns within our ICM patient population over the 6 years study period. This allowed us to reveal a typology and to quantify the proportions of different clusters of patients: light service use/Critical time intervention (58.0%), transition to regular ambulatory care (11.3%), partial transition to ambulatory care (14.4%), alternative to hospitalization (10.4%), continued ICM (4.9%) and long hospital stays (1.1%). Results overall suggest that long-term interventions were frequently not necessary and that a time-limited program was likely efficient and effective in the majority of situations or, at least, was sufficient to stabilize the situation and/or engage the patient in care.

Cluster 1 highlights the fact that, in the majority of situations (58%), ICM consists of an intervention lasting a few weeks, after which patients are rarely hospitalized and do not resort to further institutional general ambulatory care. These may be crisis situations that can be quickly overcome with ICM. In clusters 2, 3 and 4, the ICM allowed for a transition to institutional general ambulatory care and corresponds with typical intensive case management intervention characteristics. On the one hand, those in cluster 2 remain in contact with these outpatient services over time. On the other hand, in cluster 3 and 4, the use of other institutional ambulatory care tends to decrease over time. In our analyses, the only factor that differentiates these two clusters from cluster 2 is the fact that there is more non-accidental self-injuries and other mental and behavioural problems but less cognitive problems in cluster 3 and 4. Patients in cluster 3 and 4 did not differ in any clinical characteristic but patients in cluster 4 used more ICM and less general ambulatory car and hospitalization than patients of cluster 3. Patients in cluster 5 required continued ICM intervention throughout the observation period which resembled more of an ACT-type intervention. This confirms the fact that a small number of situations cannot be referred by the ICM to other ambulatory services. It may also highlight successful alternatives to long-term hospitalisation. Indeed, while the ICM makes it possible to maintain a link with patients and limit hospital admissions, their capacity to engage in general ambulatory care is not sufficient. In cluster 6, recourse to hospitalization remains necessary despite the intervention of the ICM and may represent failures of alternatives to hospitalisation. Only five patients were part of this cluster. They had a combination of clinical and social difficulties: substance abuse, poor adherence to medication, relational difficulties and housing problems. Given the complexity of these situations, the 5 year observation period may not be sufficient for the development and implementation of a care plan that would allow for stabilisation of their state of health and to avoid further hospitalizations. In addition, the ambulatory care offered may not meet the needs of this population. Finally, in cluster 3, patients were rarely hospitalized and did not readily access general ambulatory care. We assume that the ICM intervenes in these situations because this population tends not to engage in or access care rather than because they are heavy-users of care. Indeed, poor access to care could lead to a deterioration in both mental and physical health of these patients. This hypothesis could also explain the fact that the majority of deaths that occurred during the study concerned this cluster. The assumption of a population that is more difficult to engage could also explain the fact that the use of the ICM decreases over time without these patients being referred to general ambulatory services because of potential drop-outs.

Alternative to hospitalization is a key goal of ACT. The presence of a cluster relatable to this (Cluster 4) suggests this is also achieved by ICM. A small number of situations however required a sustained and long-term investment and did not always allowed for a reduction in the need for hospitalisation (Cluster 6). In several instances, a large amount of resources was allocated to a small minority of patients, a finding very similar to a previous study on hospital use only (Golay et al., 2019a). The so-called ‘Pareto-principle’ is observable in a variety of contexts and service use in ICM seems to be no exception. The small cluster of very long hospital stays was also highlighted in a previous study which stated that the question of the cost of care for people who suffer from long hospitalization is “a long-standing issue, which involves those who favored the opening of special asylums for these individuals to proponents of more open “boarding-out systems”. With the emergence of neuroleptics and antidepressants during the 1960s, it was believed that this debate would cease, as chronicity of disorders would disappear. However, if medical transformations and institutional changes certainly modified the face of psychiatry over the past 50 years, challenges remain that are very reminiscent of the past. (…) a number of patients (e.g. revolving door patients) do not seem to fit this new system and may still seek hospital support. Furthermore, very long hospitalizations, although uncommon, have not disappeared” (Golay et al., 2019a). In recent decades, the asylum has developed into an acute care facility that is no longer adapted to the needs of certain groups of patients who accumulate difficulties and who, because of the complexity of their situation, do not have access to other residential accommodation. In order to ensure that these patients do not unnecessarily crowd acute care beds and become homeless, long-stay and permanent-stay patients should benefit from supportive housing alternatives such as Housing First. In the present study, diagnoses of schizophrenia were frequent in the whole sample. Schizophrenia was a distinctive feature of heavy resource use when studying patterns in psychiatric hospital stays (Golay et al., 2019a). Nevertheless, no participant were diagnosed with schizophrenia in the “*Long hospital stays*” 6th cluster although the size of this group was very small. Psychiatric symptoms however are not the only reason for inpatient stay of heavy-users. Social problems could account for up to 20% of days of hospitalizations (Golay et al., 2019a). Alcohol and other drug use, problem with relationship and housing difficulties are very frequent in the “Long hospital stays” cluster. These specific aspects could help to identify this small, but resource heavy group with particularly complex needs who will require long term intensive support. This is important because shelter structures are not always able to cope with these difficulties. The rules of these structures have a limited acceptance for substance abuse. Anti-social traits, paranoid ideation, cognitive disorders, addictions or behavioural problems likely contribute to housing difficulties, preventing patients from being discharged from the hospital in the absence of a housing alternative. Indeed, in Lausanne, about 24% of patients referred to Intensive Case Management for Addiction were homeless (Morandi et al., 2017). Some patients also do not want the protection the hospital has to offer. We make the loose hypothesis that the possible excess mortality in cluster 3 (patients with partial transition to ambulatory care) could be explained because these patients were essentially homeless and living in the streets. This is difficult to confirm because these patients were rarely hospitalized and did not readily access general ambulatory care.

The Critical time intervention cluster (Cluster 1) is also a very important feature of what the ICM is trying to achieve while providing a time-limited intervention focused on critical or transitional periods. While cluster 1 was the biggest cluster, cluster 3 and cluster 5 also highlighted that time-limited intervention was not always possible.

This study suffers from some limitations. Firstly, clinical data was restricted to patients who were hospitalized at least once, therefore clinical data was not available for all participants. Secondly, service use of private practice psychiatrist or psychologist were not available. Thirdly, records showed that service use was very light for some patients which could be seen as contradictory because ICM is directed towards difficult situations. This could be either because the ICM team was successful, or at the contrary not able to engage the patient with care or that patients were finally treated elsewhere. We did not have further information to take this into account. Similarly, we did not have data on how patients were transitioned off ICM. This may have happened either in concert with other ambulatory care partners or because patients were lost to follow-up. Unfortunately, we could not distinguish these scenarios. However, since the inhabitants of the area are not very mobile, the Swiss social network is tight and we were able to track every psychiatric hospitalization and ICM ambulatory care, we can reasonably hypothesize that most patients transitioned off ICM were neither abandoned elsewhere nor hospitalized or in intensive ambulatory care during the period. Fourthly, cluster analysis is not model-based, which renders statistical comparison between varying numbers of clusters more difficult. We have opted for an alternative essentially based on interpretability instead, which could in some respects appear subjective. However, we do not believe that the conclusions of the study could be substantially affected by the exact number of clusters.

### Conclusion

This study highlighted that service use of ICM patients was very heterogeneous and that very long interventions were rarely necessary: most patients will need only a critical time intervention during several months and some will need as long as 5 years to benefit from it. Very few patients will need a continued ICM intervention, and for some of them, ICM will fail to provide an alternative to hospitalization. There are several public policy implications. Firstly, our study suggests that a flexible and cost-effective approach is possible for most situations when ICM patients are actively transitioned to less intensive ambulatory alternatives after a critical period (Cuddeback et al., 2013). If every demand for ICM in our study was followed by long term interventions, about five instead of one ICM team would be necessary for the 500 patients identified per 250,000 inhabitants, which is the rate recommended for FACT teams (Veldhuizen, 2007). Secondly, however, this does not mean that ICM interventions will always necessarily remain brief: a minority of patients may need several years to achieve this goal or will need continuous ICM: about 25 patients/250,000 inhabitants seem to need continued ICM, which is a sixth of the estimated cost-effectiveness number of eligible patients for original ACT (Cuddeback et al., 2006). This suggests that a differentiated approach for subgroups of patients with various levels and durations of ICM needs while facilitating consumers’ transitions from ICM may be feasible and cost-effective. Thirdly, while many patients have benefitted from more open treatments, and while the chronifying impact of hospitals is now mostly avoided, a very small number of patients do not seem to fit this new system and may still seek long term hospital support. Very long hospitalizations, although uncommon and usually fragmented, have not disappeared (Golay et al., 2019a). Lastly, In Switzerland, ICM is only available in limited specific areas and is insufficiently funded. Psychiatric ambulatory care mostly took the shape of private psychiatrists and ambulatory clinics, which do not systematically address the needs of people with the most severe mental disorders. Models of care such as ICM but also Early Intervention (Randall et al., 2015) or Housing First (Gulcur et al., 2003), if well-funded, may represent effective alternatives to hospitalizations. These interventions could reduce resource consumption by users with high levels of needs. The reflection on alternatives to hospitalization must be pursued for the few patients whose need for hospitalisation cannot be reduced despite a durable ICM intervention.
